# Genic male and female sterility in vegetable crops

**DOI:** 10.1093/hr/uhac232

**Published:** 2022-11-19

**Authors:** Zhihua Cheng, Weiyuan Song, Xiaolan Zhang

**Affiliations:** State Key Laboratories of Agrobiotechnology, Beijing Key Laboratory of Growth and Developmental Regulation for Protected Vegetable Crops, MOE Joint Laboratory for International Cooperation in Crop Molecular Breeding, China Agricultural University, Beijing, 100193, China; State Key Laboratories of Agrobiotechnology, Beijing Key Laboratory of Growth and Developmental Regulation for Protected Vegetable Crops, MOE Joint Laboratory for International Cooperation in Crop Molecular Breeding, China Agricultural University, Beijing, 100193, China; State Key Laboratories of Agrobiotechnology, Beijing Key Laboratory of Growth and Developmental Regulation for Protected Vegetable Crops, MOE Joint Laboratory for International Cooperation in Crop Molecular Breeding, China Agricultural University, Beijing, 100193, China

## Abstract

Vegetable crops are greatly appreciated for their beneficial nutritional and health components. Hybrid seeds are widely used in vegetable crops for advantages such as high yield and improved resistance, which require the participation of male (stamen) and female (pistil) reproductive organs. Male- or female-sterile plants are commonly used for production of hybrid seeds or seedless fruits in vegetables. In this review we will focus on the types of genic male sterility and factors affecting female fertility, summarize typical gene function and research progress related to reproductive organ identity and sporophyte and gametophyte development in vegetable crops [mainly tomato (*Solanum lycopersicum*) and cucumber (*Cucumis sativus*)], and discuss the research trends and application perspectives of the sterile trait in vegetable breeding and hybrid production, in order to provide a reference for fertility-related germplasm innovation.

## Introduction

Vegetable crops are of essential importance to the human diet, not only because of their enrichment in nutritional substances such as vitamins, polysaccharides, and minerals, but also due to their high content of beneficial health components, including fiber and bioactive compounds [1]. With the rapid growth of global populations and living demands, creating vegetable varieties with high yield and superior quality is one of the urgent tasks to be undertaken by vegetable breeders in the coming decades.

**Figure 1:**
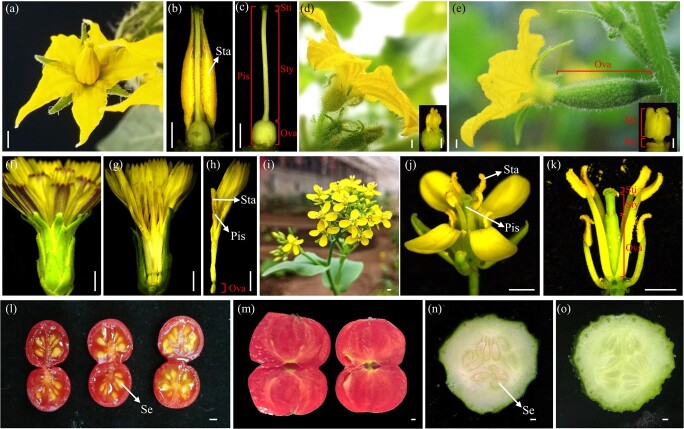
Reproductive organs of representative vegetable crops. **a**–**c** The bisexual flower of tomato (*S. lycopersicum*) (**a**) and enlarged view of male (**b**) and female (**c**) organs. **d**, **e** Unisexual flowers of cucumber (*C. sativus*): (**d**) male flower with magnified view of stamen, and (**e**) female flower with enlarged view of stigma. **f**–**h** The capitulum of lettuce (*Lactuca sativa*) (**f**, **g**), and an image of one flower (**h**). **i**–**k** Raceme of baby bokchoy (*Brassica campestris* ssp*. chinensis*) (**i**), image of one flower (**j**) and enlarged view of stamens and pistil (**k**). **l**, **m** Longitudinal views of tomato mature fruits: wild tomato accession LA1589 fruit with seeds (**l**) and a commercial cultivar showing absence of seeds (**m**). **n**, **o** Transverse sections of commercial cucumber fruits: pollinated fruit of cultivar ‘Xintaimici’ with developing seeds (**n**) and unpollinated fruit developed from parthenocarpy, with traces of aborted ovules (**o**). Sta, stamen; Pis, pistil; Sti, stigma; Sty, style; Ova, ovary; Se, seed. Scale bars = 2 mm.

Vegetable crops are generally annual herbaceous plants that predominantly propagate by seeds. Seeds are developed from embryo sacs after double fertilization—the unique mode of sexual reproduction in flowering plants. During this process, one sperm fertilizes the egg and the other sperm fuses with the central cell, which results in the formation of the embryo and endosperm, respectively [2]. Two specialized floral organs are indispensable for double fertilization: the stamen, which produces pollen, and the pistil, which bears ovules. Defects in stamen/pollen or pistil/ovules will attenuate to varying degrees male or female fertility, respectively. In vegetable crops, the morphology of reproductive organs displays great diversification. In tomato (Solanaceae) flowers, anthers joint tightly with each other to form an anther cone, which encloses the inner pistil, consisting of a stigma, a long style, and an ovary (Fig. 1a–c) [3]. Cucumber (Cucurbitaceae) cultivars mostly produce unisexual flowers: the male flower produces anthers and the female flower with inferior ovary gives rise to the fruit (Fig. 1d and e) [4]. In lettuce (Asteraceae), which is a typical self-pollinating vegetable, 25–30 flowers cluster to form a capitate inflorescence that is tightly packed by bracts (Fig. 1f and g). In each flower, anthers are fused to form the tubular androecium, a pistil formed from two carpels is located in innermost whorl, and the elongated style emerges from the tubular androecium to facilitate pollination (Fig. 1h) [5]. Flowers in baby bokchoy (Brassicaceae) are similar to those of *Arabidopsis*, with an inferior superior ovary surrounded by six stamens (Fig. 1i–k).

Hybrid seeds are widely utilized in the creation of new vegetable varieties with high yield, earliness, and growth vigor, andimproved resistance due to heterosis [6]. In most vegetable crops, the global share of hybrid seed production is increasing by 8%–10% per year [7]. However, a large proportion of vegetable crops (except Cucurbitaceae) produce morphologically tiny flowers that have strictly closed pollination (e.g. Brassicaceae, Asteraceae). Creation of male-sterile plants can save the cost of artificial emasculation and thus was commonly applied in hybrid seed production in vegetable crops such as tomato (*Solanum lycopersicum*) [8], pepper (*Capsicum annuum*) [9] and Chinese cabbage (*Brassica rapa* ssp. *pekinensis*) [10]. Intriguingly, some fruit vegetables, such as tomato (Fig. 1l and m) and cucumber (*Cucumis sativus*) (Fig. 1n and o), possess parthenocarpic ability (development of a fruit without fertilization) and thus can produce seedless fruits. Seeds generally serve as the source of hormones triggering fruit senescence, and thus the seedless trait is expected to extend the shelf life of thefruit [11]. Studies showed that seedless tomatoes contain more dry matter (except cellulose) and soluble solids than seed cultivars [12]. In cucumber, seedless pickling gherkin is more attractivefor consumers for its crunchy, firmer, and fleshier taste than the seeded variety [13]. Taken together, seedless fruit, partially derived from female sterility, is a valuable trait in fruit vegetables, not only for producers, but also for consumers [14].

Therefore, deep understanding of male and female sterility regulation is of significant importance for effective utilizationof sterile plants for hybrid seed and seedless fruit production in vegetable crops. This review will focus on genic male and/or female sterility-related mutants and the underlying genes in tomato, cucumber, and other vegetable crops (Table 1), and discuss the application perspectives of the sterile trait in vegetable production and hybrid breeding.

**Table 1:** Summary of gene information mentioned in this article.

**Gene**	**Ortholog in *Arabidopsis***	**Function(s)**	**Genetic modification**	**Phenotype(s) mainly in reproductive organs**	**Species**
*TAP3/STAMENLESS(Pr/LA0269)*	*AP3*	MADS-box transcription factor: petal and stamen identity	Ds insertion/Pr: SNP /LA0269: chromosomal rearrangement in promoter	Carpelloid stamens	*S. lycopersicum*
*TM6/MS15(26/47)*	*AP3*	MADS-box transcription factor: stamen identity	RNAi/SNP(12.7 kb deletion including promoter and first four exons)	Carpelloid stamens	*S. lycopersicum*
*SlGLO1/TPIB*	*PI*	MADS-box transcription factor: petal and stamen identity	RNAi	Disorganized stamen development	*S. lycopersicum*
*SlGLO2/TPI*	*PI*	MADS-box transcription factor: petal and stamen identity	RNAi	Disorganized stamen development	*S. lycopersicum*
*CUM26*	*PI*	MADS-box transcription factor: petal and stamen identity	15 amino acids deletion	Homologous conversion and distinct phenotype in male and female flowers	*C. sativus*
*SpAP3*	*AP3*	MADS-box transcription factor: stamen identity and sex determination	RNAi	Homeotic transformations of stamens into carpels in male flowers	*S. oleracea*
*SpPI*	*PI*	MADS-box transcription factor: stamen identity and sex determination	RNAi	Homeotic transformations of stamens into carpels in male flowers	*S. oleracea*
*TAG1*	*AG*	MADS-box transcription factor: stamen and carpel identity	Antisense RNAi	Homologous conversion and indeterminacy of the floral organs	*S. lycopersicum*
*CUM1*	*AG*	MADS-box transcription factor: stamen and carpel identity	Cosuppression by *CUM1*ectopic expression	Homologous conversion and distinct phenotype in male and female flowers	*C. sativus*
*SpAG*	*AG*	MADS-box transcription factor: microsporangial development in males and in meristem determination in females	RNAi	Indeterminate flowers, loss of stamens or carpel	*S. oleracea*
*TM5*	*SEP3*	MADS-box transcription factor: floral organ and fruit development	Antisense RNAi	Sterile stamens and ovaries	*S. lycopersicum*
*TM29*	*SEP1/2*	MADS-box transcription factor: floral organ and fruit development	Antisense RNAi	Sterile stamens and ovaries	*S. lycopersicum*
*CsSEP2*	*SEP1/2*	MADS-box transcription factor: floral organ and fruit development	SNP	Female sterility	*C. sativus*
*PsEND1*	*END1*	A pea anther-specific gene expressed in anther primordium	Ectopic expression of *barnase* driven by promoter PsEND1	Male sterility and parthenocarpic fruit	*S. lycopersicum*
*SlSES*	*SPL/NZZ*	Putative transcription factor: male and female sporogenesis	13-bp deletion	Male and female sterility	*S. lycopersicum*
*CsSPL*	*SPL/NZZ*	Putative transcription factor: male and female sporogenesis	Antisense RNAi	Reduced male and female fertility	*C. sativus*
*MS10(35)*	*DYT1*	bHLH transcription factor: microsporogenesis and tapetum development	Insertion in the promoter region and CRISPR/Cas9	Male sterility	*S. lycopersicum*
*Msc-1*	*DYT1*	bHLH transcription factor: microsporogenesis and tapetum development	7-bp deletion	Male sterility	*C. annuum*
*MS32*	*bHLH10/89/91*	bHLH transcription factor: microsporogenesis and tapetum development	SNP	Male sterility	*S. lycopersicum*
*ClATM1*	*bHLH10/89/91*	bHLH transcription factor: microsporogenesis and tapetum development	Deletion	Male sterility	*C. lanatus*
*BrTDF1*	*TDF1*	bHLH transcription factor: tapetum development	Unknown mutation in Wucai accession with abnormal tapetum	Male sterility	*B. rapa* ssp*. chinensis* var. *rosularis* Tsen
*Msc-2*	*MS1*	Plant Homeo Domain (PHD)-finger transcription factor: microspore and tapetum development	1 bp deletion	Male sterility	*C. annuum*
*CmMS-5*	*AMS*	bHLH transcription factor: microspore and tapetum development	Deletion and SNP in promoter (possible)	Male sterility	*Cucumis melo*
*CaAMS*	*AMS*	bHLH transcription factor: microspore and tapetum development	VIGS-RNAi	Male sterility	*C. annuum*
*CsWOX1/Mf*	*WOX1*	WUSCHEL-related homeobox (WOX) transcription factor: male and female sporogenesis	1 bp deletion	Male and female sterility	*C. sativus*
*SPFF*	*CEPR1/XIP1*	Leucine-rich repeat receptor-like protein kinase: pleiotropic function including anther development	2 bp deletion	Male sterility and other vegetative phenotypes	*S. lycopersicum*
*SlMAPK4*	*MAPK4*	Mitogen-activated protein kinase: signal transduction in pollen development of the binucleate stage	RNAi	Male sterility: pollen abortion	*S. lycopersicum*
*SlMAPK20*	*MAPK20*	Mitogen-activated protein kinase: signal transduction in pollen development at uninucleate-to-binucleate transition of microgametogenesis	RNAi and CRISPR/Cas9	Male sterility: pollen abortion	*S. lycopersicum*
*SlPIF3*	*PIF3*	bHLH transcription factor: pollen mitotic division	CRISPR/Cas9	Male sterility: pollen abortion	*S. lycopersicum*
*SlMYB33*	*MYB33*	R2R3-MYB transcription factor: pollen development at the mature stage	RNAi	Male sterility: pollen abortion	*S. lycopersicum*
*SlGLT1*	*GLT1*	Glutamate synthase: auxin biosynthesis in pollen development	CRISPR/Cas9	Pollen defect	*S. lycopersicum*
*SlCWIN9*	*CWIN9*	Cell wall invertase: carbon metabolism and sugar signaling in pollen development	CRISPR/Cas9	Pollen defect	*S. lycopersicum*
*CsSUT1*	*SUT1*	Sucrose transporter: sucrose support for anther and pollen development	RNAi	Male sterility	*C. sativus*
*CsHT1*	*HT1*	Hexose transporter: metabolic activity of pollen tubes	RNAi	Reduced pollen germination and pollen tube growth	*C. sativus*
*LeGWD*	*GWD*	α-Glucan, water dikinase: starch phosphorylation in pollens	Transposon Insertion	Male sterility	*S. lycopersicum*
*SAMDC1/2/3*	*SAMDC*	*S*-Adenosylmethionine decarboxylase family: reproductive development	RNAi	Male sterility	*S. lycopersicum*
*SlPIN8*	*PIN8*	Auxin transporter: auxin homeostasis in pollens	RNAi	Pollen abortion	*S. lycopersicum*
*SlFIGL1*	*FIGL1*	AAA-ATPase: male meiosis	CRISPR/Cas9	Self-sterility	*S. lycopersicum*
*CsMS-3*	*MMD1/DUET*	PHD-finger transcription factor: microsporogenesis	SNP	Male sterility	*C. sativus*
*PS-2*	*ADPG1*	Polygalacturonase protein: septum and stomium degeneration, and anther dehiscence	SNP	Non-dehiscent anthers	*S. lycopersicum*
*SlCER6*	*CER6*	3-Ketoacyl-CoA synthase family: tapetum degradation and microgametogenesis	Insertion	Non-dehiscent anthers	*S. lycopersicum*
*Style2.1*	*PRE1/BNQ*	bHLH transcription factor: cell elongation in style	Deletion in promoter	Exserted stigma	*S. lycopersicum*
*SlDELLA*	*GAI*	Putative transcription factor: restraining growth in reproductive organs	Antisense RNAi	Exserted stigma	*S. lycopersicum*
*CsGA20ox*	*GA20ox*	GA biosynthetic enzyme: promoting growth in reproductive organs	Ectopic expression	Exserted stigma	*S. lycopersicum*
*SlLst/SlETR5*	*EIN4*	Ethylene receptor protein: cell division and differentiation in style	SNP	Exserted stigma	*S. lycopersicum*
*SlIAA9*	*IAA9*	Aux/IAA transcription factor: pleiotropic function including inhibition of precocious growth of the ovary	SNP	Precocious growth of the ovary	*S. lycopersicum*
*SlPIN4*	*PIN4*	Auxin transporter: preventing precocious fruit development without pollination	RNAi	Precocious growth of the ovary	*S. lycopersicum*
*SmARF8*	*ARF8*	Auxin response factors: negatively regulating fruit initiation	RNAi	Precocious growth of the ovary	*S. melongena*
*SlARF6/8*	*ARF6*/*8*	Auxin response factors: pleiotropic function including transmitting tract formation	Silencing by Arabidopsis MIR167a	Female sterility: no pollen tube extension in pistils	*S. lycopersicum*
*CsSPT*	*SPT*	bHLH transcription factor: cell differentiation in style transmitting tract	CRISPR/Cas9	Reduced female fertility	*C. sativus*
*CsALC*	*ALC*	bHLH transcription factor: ovular guidance for pollen tubes	CRISPR/Cas9	Reduced female fertility	*C. sativus*
*CsRALF4/19*	*RALF4/19*	Cysteine-rich peptide: pollen tube integrity and ovular guidance	CRISPR/Cas9	Male sterility and reduced female fertility	*C. sativus*
*CsPID*	*PID*	Serine/threonine protein kinase: pleiotropic function including ovule initiation	SNP	Female sterility	*C. sativus*
*IMA*	*MIF2*	Mini zinc finger (MIF) protein: inhibition of cell proliferation and promotion of cell differentiation in ovules	Antisense RNAi	Female sterility and seedless fruit	*S. lycopersicum*
*Pat-k/SlAGL6*	*AGL6*	MADS-box transcription factor: ovule formation	Retrotransposon insertion	Abnormal ovule formation and reduced seed set	*S. lycopersicum*
*SlyAGL11*	*AGL11/STK*	MADS-box transcription factor: seed coat formation	RNAi	Ovule abortion and seedless fruit	*S. lycopersicum*
*SlGAMYB1*/*2*	*GAMYB1/2*	R2R3-MYB transcription factor: embryo sac development	Silencing by SlMIR159	Female sterility	*S. lycopersicum*
*PF1/SlHB15A/Pat/Pat-1*	*HB15A*	Class III homeodomain leucine-zipper (HD-ZipIII) transcription factor: preventing fruit set in the absence of fertilization	SNP and silencing by miR166	Aberrant ovule development and parthenocarpic fruit	*S. lycopersicum*
*SlCOI1*	*COI1*	Protein containing Leu-rich repeats and a degenerate F-box motif: embryo sac development	6.2-kb deletion	JA-insensitive and female sterility	*S. lycopersicum*
*SlMYB21*	*MYB21*	R2R3-MYB transcription factor: embryo sac development	TILLING and CRISPR/Cas9	Female sterility	*S. lycopersicum*
*ScRALF3*	*RALF3*	Rapid Alkalinization Factor (RALF) family: pollen and embryo sac development	RNAi	Reduced female fertility	*Solanum chacoense*
*SlCHS*	*CHS*	Chalcone synthase: flavonoids biosynthesis in reproductive organs	RNAi	Reduced female fertility and parthenocarpic fruit	*S. lycopersicum*
*uORF of SlGGP*	*GGP/VTC2*	Regulator of ascorbate synthesis: redox homeostasis in plant development including pollen fertility	SNP and CRISPR/Cas9	Male sterility and seedless fruit	*S. lycopersicum*
*SlHAK5*	*HAK5*	High-affinity K^+^ transporter: pollen K^+^ uptake and viability	CRISPR/Cas9	Reduced pollen germination and pollen tube growth	*S. lycopersicum*
*eIF4E*	*eIF4E*	Cap-binding protein constitutes the eIF4F complex: postmeiotic development of microsporocytes and tapetum	CRISPR/Cas9	Male sterility and virus resistance	*C. melo*
*ClMS1*	*HSP70*	Unknown functional data	SNP	Male sterility	*C. lanatus*
*SlSTR1*	*STR1*/*MST1*	Strictosidine synthase: pollen development	CRISPR/Cas9	Male sterility	*S. lycopersicum*
*SlEZ2*	*CLF*	Polycomb protein containing SET-domain: pleiotropic function including fruit and seed development	CRISPR/Cas9 (Cas9 driven by PPC2 promoter)	Reduced seed number	*S. lycopersicum*
*Ms-cd1*	*SIED1*	EIN3 target gene: pollen development	Higher expression in male-sterile lines	Male sterility	*Brassica oleracea*

## Male sterility

Stamens are composed of filaments and anthers, and the latter contain diploid cells that undergo meiosis to form haploid microspores that subsequently differentiate into the male gametophytes, also known as pollen grains [15]. Male-sterile mutants include plants defective in anther morphology, microsporogenesis, pollen development, and pollen function [16]. Correspondingly, male sterility is classified into structural, sporogenous, and functional types [8].

### Structural male sterility

Structural male sterility is caused by defects in stamen morphology. Mutants of this type display deformed stamens with rare pollen production [8]. Genes involved in this process are mostly stamen identity genes, such as B-, C- and E-class floral patterning genes. According to the ABC model in *Arabidopsis*, the stamen is controlled by the B-class [*AP3* (*APETALA 3*) and *PI* (*PISTILLATA*)] and C-class [*AG* (*AGAMOUS*)] genes [17, 18]. E-class *SEP* (*SEPALLATA*) genes act as co-factors with B- and C- homeotic genes in specifying stamen primordia [19]. Therefore, mutations in B-, C-, or E-class genes will result in deformed stamens in vegetable crops.

**Figure 2:**
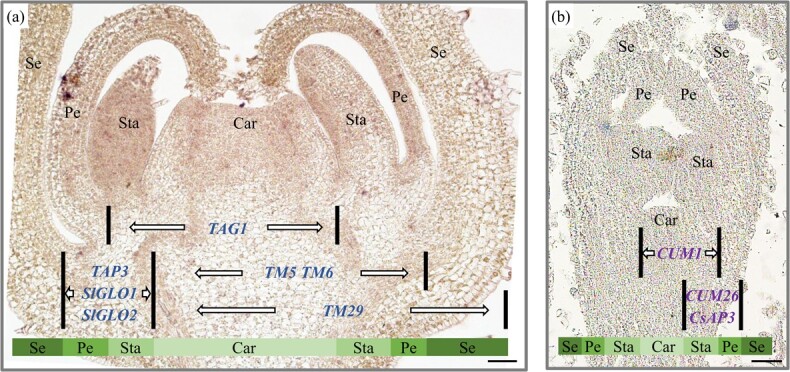
Expression patterns of reported genes involved in floral organ determination in tomato and cucumber. **a** Longitudinal section of a tomato floral bud at stage 6 [20]. **b** Longitudinal section of a cucumber floral bud at stage 6 [4]. For each gene, the area between two black lines represents its expression regions. For example, *TAG1* is expressed in stamen and carpel primordia in tomato. Car, carpel; Sta, stamen; Pe, petal; Se, sepal. Scale bars = 100 μm.

In tomato, *TAP3* and *TM6* are the homologs of the *Arabidopsis* B-class *AP3* gene. The expression pattern of *TAP3*, *TM6*, and other identity genes is indicated in Fig. 2a. Homeotic transformations of petals and stamens upon mutation of *TAP3*/*STAMENLESS* led to defective stamens with no pollen [21–23]. Similarly, *TM6* RNA interference (RNAi) plants showed carpelloid stamens [22]. In 2019, *TM6* was revealed to be the candidate gene for tomato *male sterile15(26/47)* mutants [24]. As for the other B-class gene *PI*, there are also two homologs in tomato: *SlGLO1*/*TPIB* and *SlGLO2*/*TPI*. Stamens of single *SlGLO*-RNAi lines were weakly affected, while double RNAi mutants displayed complete homeotic conversion of stamens into additional carpel-like organs and thus loss of male fertility [25]. Later, *SlGLO2* was demonstrated to be the candidate gene of natural male-sterile mutants *sl-2* and *7b-1* [26, 27]. *TM5* is one of the E-class *SEP* genes in tomato, and RNAi of *TM5* led to a series of morphologic changes of floral organs, including an altered number of organs per whorl, defective petals and styles, and sterile anthers and carpels [28]. *TM29* is another tomato *SEP* gene, and the RNAi mutants exhibited infertile stamens and ovaries, causing parthenocarpic fruit [29]. In cucumber, three ethylene biosynthesis-related genes, *F* (*Female*: *CsACS1G*) [30, 31], *M* (*Monoecious*: *CsACS2*) [32], and *A* (*Androecious*: *CsACS11*), specify the bisexual floral bud developing into unisexual flowers, and ethylene was shown to promote female flower development [33]. *CUM26* (*CUCUMBER MADS 26*) is the homolog of *PI* in cucumber (Fig. 2b). The *cum26* mutants, also called *green petals* (*gp*), displayed two perianth whorls of sepals in both male and female flowers. However, in *cum26* male flowers there were indeterminate flower buds or carpels replacing stamens in the third whorl, while in the female flower no obvious phenotypic changes were observed in whorl 3 and whorl 4, suggesting that the function of floral organ identity genes may be sex-dependent in cucumber [34]. In 2016, cucumber AP3 (CsAP3) (Fig. 2b), rather than *Arabidopsis* AP3, was found to directly bind to the promoter of ethylene receptor *CsETR1* (*ETHYLENE RESPONSE 1*) and activate its expression, an essential player for stamen arrest in female flowers, suggesting the novel characteristics of *CsAP3* in regulating female flower development in cucumber [35]. In addition to specifying stamen identity, spinach (*Spinacia oleracea*) *SpPI* and *SpAP3* are required for appropriate organ numbers, whorl development, and sex determination [36]. In lettuce, with a capitate inflorescence, there was no genetic data about stamen or carpel identity genes; however, similar expression patterns of ABC genes in lettuce suggested conserved regulatory mechanisms in floral organ development [5]. Furthermore, there have been studies to generate male sterility using the cytotoxic gene *barnase* driven by anther-specific promoter *PsEND1* (*Pisum sativum ENDOTHECIUM 1*), and the resulting anther ablation triggered parthenocarpy and seedless fruit in both miniature tomato cultivar ‘Micro-Tom’ and commercial cultivar ‘Moneymaker’, indicating the link of structural male sterility or stamen inhibition with parthenocarpic fruit development in tomato [37, 38].

### Sporogenous male sterility

**Figure 3:**
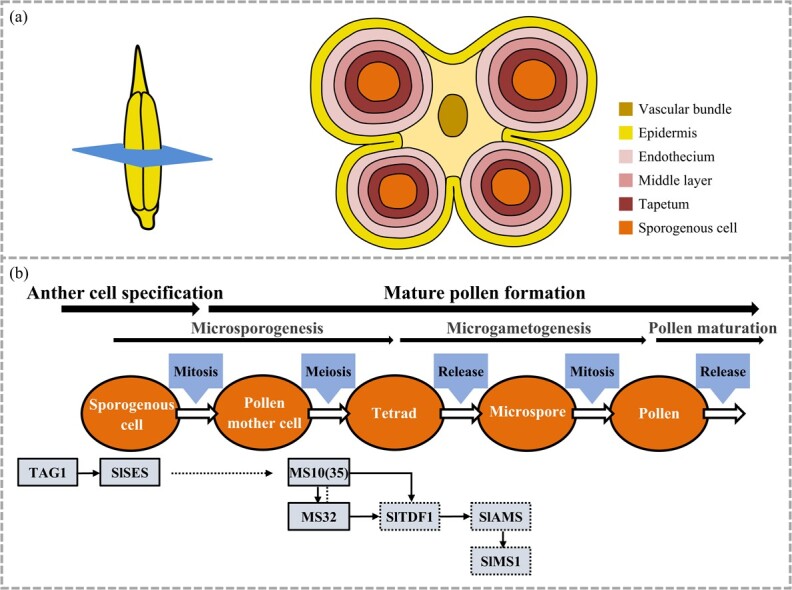
Gene regulatory networks of anther and pollen development in tomato. **a** Transverse view of a tomato anther at premeiotic stage. **b** Diagram of anther and pollen developmental stages, and the putative gene regulatory network. Solid frames: genes inferred from functional data with phenotypic effects. Dotted frames: gene function based on indirect data. Solid arrows: genetic relationships. Dotted arrow: presumed genetic relationship. Dotted line: presumed protein interaction.

Sporogenous male-sterile mutants exhibit almost normal floral morphology, but are unable to produce functional pollen, or the pollen is defective [8]. In flowering plants, pollen development includes three processes: microsporogenesis, microgametogenesis, and pollen maturation [39]. During microsporogenesis, sporogenous cells differentiate into microspore mother cells after mitosis, and then generate tetrads through meiosis [39]. In pre-meiotic anthers, the differentiated somatic cells are epidermis, endothecium, middle layer, tapetum, and sporogenous cells from outside to inside (Fig. 3a and b) [40].

The tomato γ-ray mutant *sexual sterility* (*Slses*) produced incomplete ovules and wilted anthers devoid of pollen grains, and thus loss of both male and female fertility [41]. *SlSES* encodes *Arabidopsis* SPL/NZZ (SPOROCYTELESS/NOZZLE) homologous protein and is essential for anther primordium formation in tomato (Fig. 3b) [41]. In *Arabidopsis*, SPL/NZZ is involved in sporogenesis, and this process is directly activated by C-function AG, which specifies stamen and carpel primordia development [42]. The *spl* mutant was unable to produce sporogenous cells or tapetal tissue, displaying deformed nucellus and anther wall [43, 44]. Cucumber *SPL* (*CsSPL*) is functionally equivalent to tomato and *Arabidopsis SPL*. Knockdown of *CsSPL* damaged male fertility and inhibited ovule development [45]. In tomato *MS10(35)* anthers, pollen mother cells were degenerated and failed to produce tetrads, microspores, and pollen grains, due to dysfunctional meiosis and an abnormal tapetum (Fig. 3b) [46]. *MS10(35)* is the *Arabidopsis DYT1* (*DYSFUNCTION TAPETUM1*) homolog in tomato and is essential for chromosome segregation at meiosis anaphase I and programmed cell death of the tapetum during microsporogenesis [46]. *MSC-1*, the *DYT1* homolog in pepper (*C. annuum*), exerted a similar function in male fertility [47]. Both tomato *ms32* mutants and watermelon (*Citrullus lanatus*) *Se18* mutants displayed pollen mother cell and tapetum defects [48, 49]. Their candidates, tomato MS32 (Figure 3b) and watermelon ClATM1 (Abnormal Tapetum 1), were revealed to be the homolog of AtbHLH10/89/91, which can form a protein complex with DYT1 to function synergistically in anther differentiation in *Arabidopsis* [48–50]. DYT1 can upregulate the tapetum-expressed genes *AMS* (*ABORTED MICROSPORES*) and *MS1* (*MALE STERILE 1*) in *Arabidopsis* [51]. In *ams* and *ms1* mutants, tapetal cell abnormalities were obvious, and defective pollen development appeared immediately after microspore release from the tetrads [52, 53]. Another tapetum-specific transcription factor, TDF1 (DEFECTIVE IN TAPETAL DEVELOPMENT AND FUNCTION 1) acts downstream of DYT1 and directly promotes *AMS* expression in *Arabidopsis* [54, 55]. *BrTDF1* of Wucai (*B. rapa* ssp*. chinensis* var. *rosularis* Tsen) was able to complement the tapetum and microspore defect in *Arabidopsis tdf1* mutant [56]. In tomato, the expressions of *SlTDF1*, *SlAMS*, and *SlMS1* were all significantly downregulated in *ms10(35)* and *ms32* mutants, combined with the reduced expression of *MS32* in *ms10(35)* mutants and the unchanged *MS10(35)* transcriptional level in *ms32* mutants, indicating a conserved regulatory pathway involving TAG1-SlSES-MS10(35)-MS32-TDF1-AMS-MS1 related to sporogenous male fertility in tomato (Fig. 3b) [46, 48]. By a forward or reverse genetics strategy, *MS1* homolog *MSC-2* (*CaMS1*) in pepper [57] and *AMS* homologs *MS-5* (*CmAMS*) in melon [58] and *CaAMS* in pepper [59] have been characterized, and all these mutants showed a conserved male sterile phenotype.

Some genes participate in sporogenesis development by regulating the above-mentioned essential genes. For example, the cucumber CsWOX1 (WUSCHEL-RELATED HOMEOBOX 1) transcription factor was shown to interact with CsSPL and regulate sporogenesis through the NZZ/SPL-DYT1-AMS-MS1 pathway (Fig. 5a) [60]. Tomato receptor-like protein kinase XIP1 (XYLEM INTERMIXED WITHPHLOEM 1) plays an essential role in male fertility, and its loss of function mutant *spff* (*small parthenocarpic fruit and flower*) showed shrunken anther locules with very few inviable pollen grains [61]. Some genes controlling meiotic progression also affect plant fertility. *FIGL1* (*FIDGETIN-LIKE 1*) was identified in *Arabidopsis* for its anticrossover role during meiosis, and mutation of *FIGL1* resulted in at least a 25% increase in recombination in *Arabidopsis* hybrid [62], while rice *Osfigl1* mutants displayed male sterility, with abnormal chromosome behavior and pollen abortion in the anther [63]. Tomato *Slfigl1* mutants were found to be sterile and seedless [64]. *Arabidopsis MMD1/DUET* (*MALE MEIOCYTE DEATH1*) is required for male meiotic chromosome organization and progression [65]. Cucumber *ms-3*, caused by a single-nucleotide polymorphism (SNP) in *MMD1* homolog, could not form a tetrad or microspores, thus resulting in pollen absence and male sterility [66].

After release from the tetrad, the microspore undergoes an unequal mitosis to form a large vegetative cell and a small generative cell; subsequently, the latter gives rise to two sperm cells via mitotic division, a process called microgametogenesis [67] (Fig. 3b). In tomato, SlMAPK4 (MITOGEN-ACTIVATED PROTEIN KINASE 4), SlMAPK20, and SlPIF3 (PHYTOCHROME INTERACTING FACTOR 3) have been demonstrated to play critical roles in microgametogenesis and specifically regulate post-meiotic pollen development through the sugar and auxin metabolism and signaling pathway [68–70]. Knockdown of *SlMAPK20* or *SlMAPK4* resulted in abnormally degraded cytoplasm at the binucleate stage of pollen development [68, 69]. SlPIF3 mediates the pollen mitosis I process by directly promoting the expression of both *SlGLT1* (*GLUTAMATE SYNTHASE 1*) and *SlCWIN9* (*CELL WALL INVERTASE 9*) in the tomato anther [70]. Knockout of *SlGLT1*/*SlCWIN9* phenocopied the defective bicellular pollen phenotypes of *Slpif3* mutants [70]. Tomato *SlMYB33* was shown to function in the pollen maturation process. Pollen grains in *SlMYB33*-RNAi plants displayed slight irregularity during the binucleate stage and collapse and shrinkage at the mature stage, and thus exhibited aberrant pollen viability and poor male fertility [71]. Plant metabolites, like saccharides and polyamines, can affect male gamete development as well. Cucumber SUT1 (SUCROSE TRANSPORTER 1) regulates anther and pollen development by ensuring carbohydrate supply [72]. *CsSUT1-*RNAi plants showed shriveled pollens and male sterility [72]. Downregulating cucumber *HT1* (*HEXOSE TRANSPORTER 1*) reduced pollen germination and tube growth [73]. In tomato, mutation of the gene for α-glucan water dikinase (GWD), a key enzyme controlling starch degradation, led to pollen grains accumulating excess starch and male gamete lethality, with only 0.4% paternal transmission [74]. The reduction of polyamines in tapetal tissue, executed by knocking down the biosynthesis-related gene *SAMDC1*/*2*/*3* (*S-ADENOSYLMETHIONINE DECARBOXYLASE*) under the tapetal-specific A9 promoter, led to shrunken and distorted pollen grains and male sterility [75]. In addition, auxin homeostasis is crucial for reproductive development. Knockdown of auxin efflux transport PIN8 (*PIN-FORMED 8*) resulted in decreased auxin content and ~80% of pollen grains with abnormality and lack of viability [76].

### Functional male sterility

In flowering plants, after a successive process of cell degradation in the tapetum, septum, and stomium, mature pollen is released from the anther [77]. In functional male-sterile mutants, the pollen is normal but cannot reach the stigma or cannot germinate on the stigma [8]. To some extent, diclinism in most cucurbit crops (e.g. cucumber) is a kind of natural functional male sterility due to the positional separation of the stamen (in the male flower) and pistil (in the female flower). Manual pollination can restore the fertility of this functional type of male sterility.

**Figure 4:**
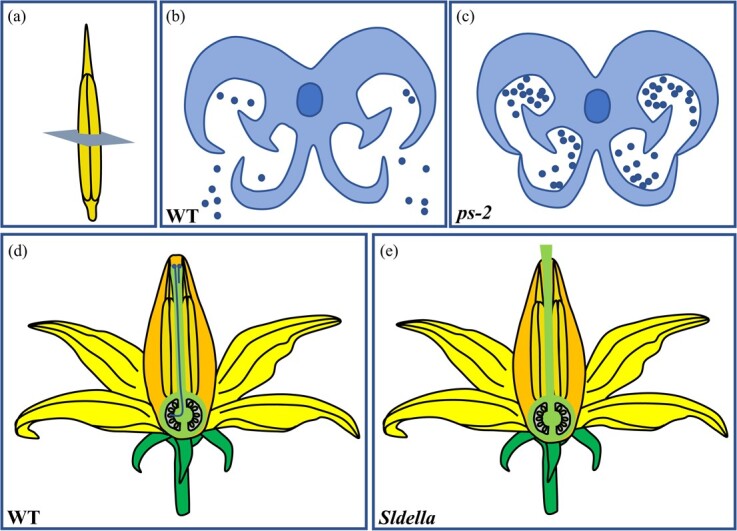
Two types of functional male sterility in tomato. **a** Schematic drawing of tomato stamen. **b** In wild-type tomato, dehiscence of mature anthers gives rise to release of pollen grains. **c** In the *ps-2* mutant, indehiscence of mature anthers blocks pollen grain dispersal. **d** In wild-type tomato, pollen grains adhere to the stigma and germinate, then pollen tubes extend along the transmitting tract towards ovules. **e** In the *Sldella* mutant the exserted style prevents pollen grains landing on the stigma, leading to pollination failure.

In tomato, spontaneous mutants *ps* (*positional sterile*) and *ps-2* conferred functional male sterility due to unsplit anthers (Fig. 4a–c). A *polygalacturonase* gene (*PG*), the homolog of *Arabidopsis ADPG* (*ARABIDOPSIS DEHISCENCE ZONE POLYGALACTURONASE*), is responsible for the tomato *ps-2* male-sterile phenotype. PGs belong to one of the largest hydrolase families and are associated with a wide range of plant developmental programs, including anther dehiscence [78]. In breeding practice, *ps-2* has been utilized for tomato hybrid seed production in Eastern Europe due to its low level of self-pollination and availability of viable pollen by manual breeding [79]. Moreover, mutation in tomato β-ketoacyl-coenzyme A synthase *SlCER6* (*ECERIFERUM 6*), which is required for the biosynthesis of very-long-chain fatty acids, led to defective cuticle formation during anther development. Thus, *Slcer6* mutants showed abnormal tapetum development and inhibited pollen dispersal [80]. *Slcer6* mutants displayed two times lower seed production than in wild-type self-crossing when used as the pollen donor. Considering the impaired microgametogenesis, *Slcer6* mutants exhibited the characteristics of both sporogenous male sterility and functional male sterility [81].

Besides non-dehiscent anthers, an exserted stigma also leads to self-pollination failure because pollen can hardly reach the stigma properly (Fig. 4d and e). Style length was previously found to be associated with allogamy/autogamy (cross-pollination/self-pollination) [82]. *Style2.1* accounts for the stigma exsertion in wild, allogamous species, and a deletion of 450 bp in the promoter of *Style2.1* was responsible for the transition of long style to short style, promoting the evolution of self-pollination in cultivated tomatoes [82]. The phytohormone gibberellin (GA) was found to regulate style length by promoting cell expansion and proliferation. Knockdown of the GA signaling protein gene *SlDELLA* resulted in excessively elongated style and self-sterility in tomato (Fig. 4d and e) [83]. Similarly, overexpressing citrus *GA biosynthetic enzyme GA20OX* (*GA20-OXIDASE*) in tomato caused an elongated style and protruded stigma [84]. Tomato accession T431 exhibited >95% male sterility due to stigma exsertion under higher temperature, for which the *SlLST* (*Long Styles*) gene encoding an ethylene receptor protein was the key candidate [85]. Further study showed that, unlike the stigma exsertion in wild tomato, high temperature-induced stigma exsertion was caused by more seriously shortened stamens than pistils in cultivated tomato, and exogenous jasmonate treatment could effectively rescue this type of stigma exsertion, suggesting a new function of jasmonic acid (JA) in improving plant abiotic stress tolerance [86].

Another cause of functional male sterility is inconsistent maturation of anthers and pistils or preanthesis ovaries, which refers to ovaries that can initiate development without/before pollination stimulation. In tomato, loss of function of the negative regulator of auxin response *Aux/IAA9* resulted in seedless fruits due to precocious growth of ovaries, despite the mutant pistils being fertile by hand pollination [87]. Similarly, downregulation of *SlPIN4* triggered seedless fruit because of fruit development initiated prior to fertilization [88]. In eggplant (*Solanum melongena*), the spontaneous dominant mutant *13-3* produced parthenocarpic fruits with reduced expression of auxin response factor *SmARF8* (*AUXIN RESPONSE FACTOR 8*). Silencing *SmARF8* by RNAi in eggplant generated seedless fruits, whereas hand-pollination of *SmARF8*-transgenic plants could produce normal, seeded fruits [89].

## Female sterility

In breeding practice, the application of female sterility is much less common than that of male sterility. Related studies are sporadic and unsystematic, mainly focusing on parthenocarpy and seedless fruit. Seeds are considered detrimental to the edible quality of the fruit, e.g. seeds in eggplant can lead to browning and bitterness of the flesh [89]. Therefore, seedless fruit is a desirable trait in many horticultural crops, e.g. triploid seedless watermelon [11], seedless citrus varieties bred from Satsuma mandarin (*Citrus unshiu*) by somatic hybridizations [90, 91], and seedless grape ‘Corinth’ and ‘Thompson’ cultivars (*Vitis vinifera*) [11]. Female fertility defects generally result from abnormalities of the ovary or ovule development.

### Weakened female fertility caused by ovary defects

The fruit is generally developed from the carpel, and loss of carpel identity genes can cause ovary developmental abnormalities and thus defects or absence of female fertility, such as the C-function genes (tomato *TAG1*, cucumber *CUM1*, and spinach *SpAG*) and E-function genes (tomato *TM5* and *TM29*, and cucumber *SEP2*) (Fig. 2a and b). In tomato transgenic plants with *TAG1* silenced, indeterminacy was detected in the fourth whorl and carpels were replaced with indeterminate floral meristems [28]. Regarding AG homologs in unisexual flowers, they seemed to remain similar in function, although the *AG*-suppressed plants exhibited unique phenotypic characteristics related to sexual dimorphism in dioecious spinach and monoecious cucumber [34, 36]. In *TM5*-RNAi tomato, the inner three whorls of the flower were all defective, and the sterile carpel with deformed style was incompletely fused [3]. Knockdown of *TM29* in tomato resulted in infertility of the ovary, with ectopic shoots growing from the deformed fruit [29]. In cucumber, mutation of *CsSEP2* led to perturbed floral development and infertility of the ovary, with an enlarged stigma and seedless fruit shed precociously from the peduncle [92].

**Figure 5:**
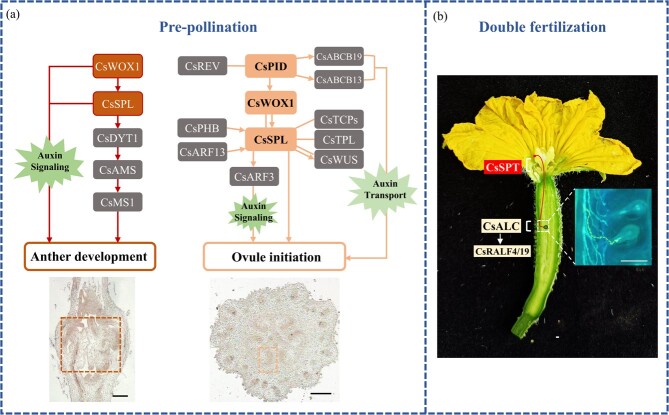
Gene regulatory networks in reproductive organs at pre-pollination and double fertilization stage in cucumber. **a** Working model involving CsPID, CsWOX1, and CsSPL in cucumber sporogenesis in cucumber. On the left is a longitudinal section of a male bud at stage 9 and on the right is a transverse section of a female bud at stage 9. Scale bars = 200 μm. **b** Functions of CsSPT and CsALC-CsRALF4/19 in pollen tube guidance in cucumber. Scale bar = 1 mm. Gray boxes represent genes with unreported phenotypic data. Short solid arrow indicates a genetic relationship; a solid line represents protein interaction. Dashed yellow line in (**b**) indicates extension of pollen tube and its entry into the ovule.

In angiosperms, successful double fertilization begins with pollen grain deposition on the stigma, adhesion, hydration, and germination to produce pollen tubes [93]. Then pollen tubes pass through the reproductive tract tissues [also known as the transmitting tract (TT)] inside the stigma, style, and ovary towards the ovules [94]. Female fertility defects can also result from abnormalities in the stigma, style, or TT that block the pollen tubes targeting the ovules. In *Arabidopsis*, genes responsible for TT development include *SPT* (*SPATULA*) [95], *ARF6/8* [96], *HEC*s (*HECATE*s) [97], *NTT* (*NO TRANSMITTING TRACT*) [98], and *HAF* (*HALF FILLED*) [99]. Downregulation of tomato *ARF6* and *ARF8* led to smaller organs and female sterility, as a result of pollen failure to germinate on the stigma or to grow along the TT in transgenic plants [100]. In cucumber, a recent study showed that CsSPT and CsALC (*Arabidopsis* ALCATRAZ homolog) functioned redundantly in maintaining female fertility. *Csspt* single mutants showed 60% lower seed set from the defective style TT, while *Csspt Csalc* double mutants showed complete female sterility due to absence of pollen tube extension channels [101]. In addition, CsALC participated in pollen tube guidance by positively regulating the expression of *CsRALF4* (*RAPID ALKALINIZATION FACTOR 4*) and *CsRALF19* in cucumber, and mutation of *CsALC* led to 95% reduction in female fertility (Cheng *et al.*, unpublished results). Therefore, during the long journey from stigma to ovules, pollen tube extension is mediated by CsSPT at the preovular guidance stage (early stage of pollen tube guidance) and by CsALC to approach ovules at the late stage in cucumber (Fig. 5b).

### Damaged female fertility derived from ovule defects

In *Arabidopsis*, ovules are located within the ovary, attached to the placenta by the funiculus. Mature ovules consist of embryo sacs, inner and outer integuments, and the funiculus. Ovule development is divided into four phases: (i) initiation of ovule primordia from the placenta; (ii) appearance of the megaspore mother cell (MMC) and its differentiation into four haploid megaspores, as well as initiation of the inner and outer integument; (iii) embryo sac formation and integument maturation, in which the most proximal megaspore undergoes three mitotic division cycles to form the seven-celled embryo sac, including two synergids, an egg, a central cell, and three antipodal cells; and (iv) fertilization and embryogenesis [102–104]. Disruption of any of the above-mentioned developmental phases will result in an ovule defect.

The cucumber auxin transport-related gene *CsPID* (*PINOID*) plays an essential role in ovule initiation. *Cspid* mutants exhibited no ovule formation in the placenta and were female-sterile [105]. CsPID interacted directly with the HD-ZIP III transcription factor CsREV (REVOLUTA), whose homolog in *Arabidopsis* is required for meristem initiation at lateral positions (Fig. 5a) [106]. In *Cspid* mutants, two auxin transport-related genes, *CsABCB19* (*ATP-BINDING CASSETTE B19*) and *CsABCB13*, as well as the ovule development-related genes *CsWOX1*, *CsSPL*, and *CsWUS* (*WUSCHEL*) were all downregulated, suggesting that they may act in the same pathway in regulating ovule initiation (Fig. 5a). Cucumber *CsWOX1* and *CsSPL* are involved in female organ development by mediating auxin signaling. *Cswox1* mutants displayed no ovule initiation and significantly reduced *CsSPL* transcript accumulation [60]. CsWOX1 could interact with CsSPL, and CsSPL could interact with CsTPL (TOPLESS) and CsTCP23 (TEOSINTE BRANCHED1/CYCLOIDEA/PCF family protein 23) (Fig. 5a) [60]. In severe *CsSPL*-RNAi lines the ovules displayed a finger-like structure without nucellus and integument [45]. Auxin was detected to be greatly decreased in the male and female organ primordia of *Cspid*, *Cswox1*, and *Csspl* mutants [45, 60, 105]*.* CsSPL stimulated the expression of *CsARF3*, and was positively regulated by CsARF13 and CsPHB (PHABULOSA) during sporogenesis (Fig. 5a) [45]. CsWOX1 may regulate sporogenesis via the CsSPL-mediated pathway and/or by modulating auxin signaling in cucumber (Fig. 5a) [60]. Homologs of *SPL* in tomato displayed similar roles in megasporogenesis. There was no nucellus (MMC) or integument development in tomato *Slses* mutants [41]. Besides auxin, the level of GAs and brassinosteroids (BRs) was reported to regulate ovule numbers in tomato by mediating ovule initiation [107, 108]. GA reduced ovule number and BR promoted ovule initiation by inhibiting GA biosynthesis in tomato [107, 108]. Tomato IMA (INHIBITOR OF MERISTEM ACTIVITY) promoted nucellus differentiation and inhibited cell proliferation by negatively regulating *WUS* expression [109]. *IMA*-RNAi plants displayed finger-shaped ovules with overgrowth of integuments or indeterminacy of ovule structure, and no MMC was formed [109]. Loss of function of *SlAGL6* (*AGAMOUS-LIKE 6*) by a retrotransposon insertion resulted in parthenocarpic fruits and defective ovules with abnormal micropyle in tomato [110]. *AGL11*, also known as *STK* (*SEEDSTICK*), controls funiculus development and seed detachment in *Arabidopsis* [111]. Its homolog in tomato, *SlyAGL11*, was reported to regulate seed coat formation and seed numbers [112]. Suppression of *SlyAGL11* led to seedless tomatoes with premature fruit set and faster fruit growth, resembling a strong parthenocarpic phenotype [112].

Even if ovule initiation is normal, embryo sac arrest also can lead to female sterility. JA has been shown to play key roles in ovule late development by analysis of mutants *jasmonate-insensitive1-1* (*Sljai1-1*; mutated in the JA coreceptor *COI1*) in tomato [113]. *SlMYB21* was greatly downregulated in *Sljai1-1* mutants, and the ovules of *Sljai1-1* and *Slmyb21* mutants showed similar phenotypes with increased callose deposition and cell vacuolation in the embryo sac, leading to premature elimination of the nucellus before fertilization and thus female sterility [113]. On the other hand, JA promotes *SlMYB21* expression, which positively feeds back into JA biosynthesis, and thus promotes ovule late development in tomato [113]. Moreover, the miR159–GAMYB1/2 and miR166–SlHB15A modules are crucial for tomato ovule development and fruit set. *SlGAMYB1*/*2* silencing by *SlMIR159* overexpression resulted in abnormal growth of the embryo sac and misregulation of pathway genes associated with ovule and female gametophyte development [114]. The mutant *pf1* (*parthenocarpic fruit 1*) produced seedless fruits, and the causal gene was identified as *SlHB15A.* Knockdown of *SlHB15A* by *miR166* led to aberrant ovules and parthenocarpic fruit [115]. The *pat* and *pat-1* tomato accessions with parthenocarpic fruits were confirmed to be alleles of *pf1* [115]*.* Recently, small peptides have been revealed to play important roles during the reproduction process in *Arabidopsis* [116], such as *RALF4/19* in pollen tube integrity [117], *RALF34* in triggering pollen tube burst [118], and *LURE*s in attracting pollen tubes to ovules [119]. Potato (*Solanum chacoense*) *ScRALF3* participates in both male and female gametophyte development. *Scralf3* mutants produced pollen grains with reduced viability and fewer seeds, primarily due to abnormal nuclear distribution and asynchronous nuclear divisions in the embryo sac [120, 121].

Besides the above, in traditional breeding, gamete sterility derived from chromosome behavior disorder during meiosis is a key application in seedless fruit production, e.g. the self-infertility of triploid watermelon and banana from gametic chromosome imbalance [122]. Chromosome translocation is another way to generate seedless fruits in watermelon and banana [123, 124]. Recently, a homozygous translocation occuring on watermelon chromosome 6 led to meiotic defects at metaphase I and thus the less seed phenotype of the *F*_1_ hybrid, providing new reference for the application of reciprocal translocation in less seed fruit breeding at diploid level [122].

## Perspectives

Fertility genes and related mechanisms are intensively explored in model plants, including *Arabidopsis* and grain crops. Due to the importance of hybrid seed production, more and more male-sterile mutants and related genes have been characterized in vegetable crops. However, the underlying mechanisms and regulatory networks remain unclear and fragmented. Besides the above-mentioned genes directly affecting fertility, more regulators have been found to participate in reproductive development in an indirect manner. Several studies have shown the close relationship of secondary metabolites and plant fertility. Silencing of tomato chalcone synthase (*CHS*), the first gene in the flavonoid pathway, caused diminished pistil fertility and parthenocarpic fruits [125]. Besides, overproduction of ascorbic acid, caused by mutation of the ascorbate synthesis regulator GGP (*GDP-L-GALACTOSE PHOSPHORYLASE*), resulted in disrupted anther and pollen development and male sterility in tomato [126]. There are other examples of indirect regulators. Knockout mutants of the high-affinity K^+^ transporter *SlHAK5* in tomato exhibited impaired pollen germination and pollen tube growth, and resultant reduced seed set, indicating the importance of K^+^ uptake during reproduction [127]. In melon, a recent study showed that mutation of *eIF4E*, encoding a cap-binding protein functioning in mRNA circularization and cap-dependent translation, led to melons with increased virus resistance and male sterility. Mutants of *eif4e* displayed postmeiotic defects in both microsporocytes and tapetum [128].

In the past decades, mining of male/female-sterile genes has relied mainly on natural or artificial mutants. With advances in plant genetic transformation and genome manipulation technology such as the CRISPR/Cas9 system, it is much easier, faster, and more precise to create mutant materials and perform functional analysis. For example, male-sterile tomato plants without exogenous vector were obtained by targeting *SlMS10* gene via CRISPR/Cas9-mediated genome editing [129]. By targeting a stamen-specific gene, the putative strictosidine synthase gene (*SlSTR1*), a novel male-sterile tomato line with abnormal pollen grains was generated [130]. Meanwhile, a transgenic maintainer was created in the background of a male-sterile line transformed with *SlSTR1* and a seedling-color marker gene (R2R3 MYB transcription factor gene *ANT1*) [130]. Offspring of crosses between a homozygous male-sterile plant and hemizygous maintainer will be half male-sterile plants and half fertile maintainer plants that can be easily identified by seedling color [130]. This biotechnology-based platform has great practical potential for hybrid seed production in diverse vegetable crops [130]. Meanwhile, the fluorescence marker gene (mCherry, GFP) and the visualized marker gene (Ruby) could also be adopted as a proxy for isolation of transgene or transgene-free vegetable crops on a large scale, which will greatly save time and workload [131]. Notably, TKC (Transgene Killer CRISPR system) technology could achieve self-elimination of the CRISPR construct and produce all-transgene-free progenies [132, 133]. In addition, iTRAQ (isobaric tags for relative and absolute quantification)-based proteomics was applied in the exploration of the protein regulation network of the male-sterile mutant *ms10(35)*, and fatty acid metabolism was speculated to be the cause of male sterility [134]. iTRAQ and PRM-based proteomics analysis showed that metabolism pathways, including sugar, lipid, and fatty acid pathways, played important roles in pollen abortion in the tomato *ms7* mutant [135].

Despite sterile mutants having advantages of decreasing cost and increasing seed purity during hybrid seed production, complete sterility (referring to both male and female sterility) is undesirable due to the difficulty in germplasm retention. An ideal male/female sterile line should be deficient in fertility, but with normal vegetative growth or fruit development, such as the widely used functional male-sterile mutant *ps-2* in tomato breeding practice. For genes with pleiotropic functions, disruption in targeted reproductive organs can be achieved via knockout driven by reproductive organ (pollen or ovule)-specific promoters. For example, knockdown of *SlIAA9* in tomato fruits using two flower-specific promoters, *Solyc03g007780* (*Psol80*) and *Solyc02g067760* (*Psol60*), resulted in parthenocarpic fruits without other defects in vegetative tissues [136]. Similarly, fruit-specific gene editing of tomato *SlEZ2*, a SET-domain-containing polycomb protein, through the Cas9 system driven by a *phosphoenolpyruvate carboxylase 2* (*PPC2*) gene promoter enabled observation of fruit phenotypes lacking additional developmental perturbations [137]. Tissue-specific gene editing will have more applications in vegetable crops to obtain male-/female-sterile mutants by targeting essential genes under reproductive organ-specific promoters.

In agricultural practice, in addition to male-sterile lines, sustainable seed production requires restorer lines and maintainer lines, which entail huge costs. The dominant genic male-sterility (DGMS) system enables an efficient cultivation mode with a 1:1 ratio of male-sterile to male-fertile plants in *F*_1_ hybrid plants without seed sorting and separation processes, and eliminates the risk of genetically modified pollen entering the ecosystem, and thus it is highly appreciated in cross-pollinated crops like maize and oilseed [138]. One of the rare examples of DGMS application in vegetable crops is the spontaneous mutant 79-399-3 in cabbage (*Brassica oleracea*), which is controlled by single dominant gene, *Ms-cd1*, and has served as the donor for the creation of dominant male-sterile lines in multiple Brassicaceae crops, e.g. cabbage, broccoli, kohlrabi, and Chinese kale [139, 140]. Map-based cloning showed that the *Arabidopsis* homolog gene *SIED1* (*SALT-INDUCED AND EIN3/EIL1-DEPENDENT 1*) was the *Ms-cd1* gene and a KASP marker was developed for rapid identification of *Ms-cd1* loci [140, 141]. Recently in watermelon, a new DGMS gene, *HSP70* (*HEAT SHOCK PROTEIN 70*), was identified as the candidate of *Clms1* mutant, providing a new gene resource for the DGMS system [142]. In addition, the fusion of cytotoxic genes to tapetum-specific promoters led to genetic male-tissue cell ablation and could be an effective biotechnological tool to generate dominant male sterility [38]. As mentioned above (see section Structural male sterility), the chimeric construct of the ribonuclease gene (*barnase*) driven by the anther-specific promoter *PsEND1* gave rise efficiently to dominant male sterility in Brassicaceae and Solanaceae [38, 143]. Some other promoters from sporogenesis-related genes, e.g. *TDF1*, *AMS*, and *MS1*, can be applied to trigger the expression of cytotoxic genes in specific male tissue to cause DGMS.

Compared with DGMS, an inducible male-sterility system is more labor-saving due to its elimination of the dependence on maintainer lines and restorer genes during hybrid seed production [144]. Chemicals such as herbicides act as common inducers, e.g. the glyphosate-mediated male sterility system developed in maize, which exploited the differential expression of the glyphosate-resistance gene in male tissue and the rest of the plant [145]. Other chemical-inducible male sterility systems are established on the basis of conversion of a non-toxic to a toxic chemical catalyzed by an anther-localized conversion gene, e.g. the l-ornithinase (*argE*) gene converted the non-toxic compound *N*-acetyl-phosphinothricin (N-ac-PPT) into the herbicide phosphinothricin (PPT) in tobacco and rice [146, 147], and the *TAP1-DMRtDAAO* gene converted d-glufosinate into l-glufosinate in tobacco [148]. In addition to chemical agents, environmental signals can also induce male sterility, such as photoperiod/thermosensitive genetic systems [also called environmentally sensitive genic male sterility (EGMS)] in rice [149, 150]. The above induction systems can be optimized and utilized in vegetable crops.

In addition to genic male sterility (GMS), cytoplasmic male sterility (CMS), caused by incompatibility of the nuclear and mitochondrial genomes, has been broadly used in hybrid breeding and genetic improvement of vegetable crops, e.g. the utilization of *Ogura* CMS, *Polima* CMS, and *nap* CMS in Chinese cabbage (*B. rapa* ssp. *pekinensis*) germplasm innovation [151, 152]. Nuclear genes that regulate mitochondrial genes relevant to CMS were called fertility restorer genes [152]. So far, many mitochondria-located CMS genes have been identified in vegetable crops, e.g. *ORF138* for *Ogura* CMS in radish (*Raphanus sativus*) [153, 154], *ORF220* in mustard (*Brassica juncea*) [155, 156], and *orf137* in tomato [157], but not all corresponding restorer genes have been identified. More information about the mechanism of CMS and its application can be found in a recent review [151].

As elite seeds derived from hybrid breeding are the core competitiveness of the vegetable industry, dissecting fertility-related genes and mechanisms can not only further our understanding of sexual reproduction, but also create more sterile plants to facilitate crossbreeding or obtain seedless fruit. In addition to traditional breeding goals (yield, disease resistance), specific traits such as nutritional or bioactive component enrichment [158], tolerance of extreme environments (e.g. high temperature, salinity), and adaptability to different cultivation modes (e.g. dense planting and soilless culture) will attract more attention during vegetable breeding in the future.

## Acknowledgements

We thank Dr Luo Chen for the pictures of lettuce flowers in Fig. 1f–h, and Dr Chen Jie for the image of a tomato floral bud section in Fig. 2. We apologize to authors not cited due to space limitations. This work was supported by National Natural Science Foundation of China (32025033) and (31930097), and the Chinese Universities Scientific Fund (2022TC009).

## Author contributions

C.Z. and S.W. wrote the original draft and prepared the figures; Z.X. supervised the work and revised the manuscript. All authors have read and agreed to the published version of the manuscript.

## Conflict of interest

The authors declared no conflict of interest.
